# Acute Tonsillitis

**Published:** 1897-07

**Authors:** George Foy

**Affiliations:** Dublin, Ireland


### ACUTE TONSILLITIS.

DUBLIN, IRELAND, June 18, 1897.

*Editor Atlanta Medical and Surgical Journal:*

On page 283 of your journal for this month you give a formula for the treatment of acute follicular tonsillitis, a disease of which I have had a rather large experience for the last twenty years, during the last ten of which I found the following a very useful perscription, to wit:

℞ Tincture of aconite .................................................. ʒ ss.

Chloroform water ...................................................℥ ij.

Distilled water to.. ...................................................℥ iv.

M. ft. mist.

A teaspoonful every five minutes for twelve doses, afterwards a dose every hour. If necessary I repeat the mixture and direct the repetition to be taken as before, beginning with five-minute doses. Yours, etc.,

GEORGE FOY, M.D.

---

Numbers of physicians throughout the country have learned with sorrow of the death of Dr. J. Lewis Smith, and Dr. William T. Lusk, both of New York. Dr. Smith was nearly seventy years of age, and for many years had been Professor of Diseases of Children in Bellevue Hospital Medical College. He was the author of a well-known work on the diseases of children, and had been a frequent contributor to the literature of pediatrics. Dr. Lusk's name has become familiar to physicians of this country, particularly from his standard treatise on the "Science and Art of Obstetrics," which appeared in 1881, and which has reached a wide popularity at home and abroad. Dr. Lusk was fifty-eight years of age, and for twenty-five years has been Professor of Obstetricts and Gynecology in Bellevue Hospital Medical College. The death of these gentlemen removes two of the most distinguished of American physicians, each of whom had been an ornament to his profession and a blessing to to his kind.

